# In Silico Dosimetric Comparison of Intensity-Modulated Photon Versus Proton Therapy for Pelvic and Para-Aortic Nodal Recurrence of Prostate Cancer Following Prior Irradiation

**DOI:** 10.3390/cancers18213452

**Published:** 2026-10-26

**Authors:** James A. Knight, Hao Chen, Anh Tran, Bethlehem Floreza, Heng Li, Aditya Halthore, Rachit Kumar, Michael Goldstein, Ana Kiess, Daniel Song, Stephen Greco, Curtiland Deville

**Affiliations:** 1 Department of Radiation Oncology and Molecular Radiation Sciences, Johns Hopkins University School of Medicine, Baltimore, MD 21205, USA; atran43@jhu.edu (A.T.); bflorez1@jhmi.edu (B.F.); hengli@jhu.edu (H.L.); ahaltho1@jhmi.edu (A.H.); rkumar16@jhu.edu (R.K.); mgolds33@jhmi.edu (M.G.); akiess1@jhmi.edu (A.K.); dsong2@jh.edu (D.S.); sgreco1@jhmi.edu (S.G.); 2 Department of Radiation Oncology, University of Florida College of Medicine, Jacksonville, FL 32206, USA; 3 Department of Radiation Oncology, University of South Florida Morsani College of Medicine, Tampa, FL 33606, USA; haoc@usf.edu

**Keywords:** prostate cancer, nodal, recurrent, re-irradiation, proton, intensity-modulated proton therapy, IMPT, pencil beam, dosimetry, dosimetric

## Abstract

**Background/Objectives:** Limited data exists comparing photon-based intensity-modulated (IMRT) or volumetric modulated arc therapy (VMAT) against intensity-modulated proton therapy (IMPT) when treating pelvic or para-aortic nodal recurrences of prostate cancer following prior irradiation. The aim of this study was to evaluate dosimetric differences between VMAT and IMPT when treating nodal recurrences in the re-irradiation setting. **Methods**: VMAT and IMPT plans for 15 patients with node-recurrent disease were evaluated. Prescription doses to the whole pelvis and prostate bed planning target volumes (PTVs) from the initial VMAT plans were 45–50.4 Gy and 66.6–72 Gy, respectively. IMPT re-irradiation prescription doses were 45–53.75 Gy radiobiological equivalent (GyE) (proton radiobiological effective dose 1.1) to at-risk pelvic and para-aortic nodal chains and 60–64 GyE to gross nodal disease. For re-planning and comparative study purposes, clinical target volume (CTV) prescription doses were standardized to 50.4 Gy for the regional pelvic and para-aortic nodes, and 63 Gy for the grossly involved nodal disease. Organ-at-risk (OAR) contours were standardized and re-irradiation plans were generated on Raystation. Student’s *t*-test was used to compare CTV coverage and dose to OARs. **Results**: CTV coverage was met for all plans, with 99% of CTVs receiving ≥99% of prescription doses. Composite analysis demonstrated IMPT not only sustained dosimetric benefits in the re-irradiation setting for all OARs except sigmoid but also conferred significant advantages. Select endpoints with significant differences on composite analysis included bowel cavity V45Gy, left kidney V18Gy and mean dose, right kidney V18Gy and mean dose, and integral dose to ‘Body-CTV’. **Conclusions**: Composite analysis of the initial treatment and re-irradiation plans showed that IMPT delivered and maintained significant OAR dose reductions in the nodal re-irradiation setting, potentially decreasing the risk of long-term toxicities. This provides a rationale for the utilization of IMPT in this clinical setting.

## Simple Summary

Among patients who have prostate cancer recurrence after having received radiation, about 20% have disease in the pelvic lymph nodes, and 15–17% have disease in the para-aortic nodes. Para-aortic nodal involvement is linked to reduced survival, but how to safely re-irradiate patients’ disease without causing severe toxicity remains a concern. In our study, 15 node-recurrent patients with prior radiotherapy for prostate cancer were re-planned to compare proton vs. photon delivery. CTV coverages were similar for all plans, while proton therapy delivered lower doses to several metrics for the bowel, kidneys, and uninvolved parts of the body. The data may help inform future delivery of pelvic and para-aortic proton therapy for prostate cancer.

## 1. Introduction

The optimal management of isolated pelvic and para-aortic nodal recurrences after definitive or salvage external beam radiotherapy (EBRT) for prostate cancer remains under investigation. For patients with clinically detected recurrence after definitive EBRT, pelvic lymph nodes are involved in 21.3% and abdominal lymph nodes in 9.1% [1]. Para-aortic nodal metastases occur in about 15–17% of patients with oligorecurrent disease, and para-aortic nodal involvement has been shown to be a significant predictor of reduced biochemical and distant metastasis-free survival [2]. In the era of positron emission tomography/computed tomography (PET/CT), isolated nodal detection has increased. Studies comparing prostate-specific membrane antigen-based PET/CTs (^68^Ga-PSMA PET/CT) against conventional imaging studies or histopathologic methods have demonstrated superior detection of pelvic and distant metastases. Approximately 31% of patients who undergo ^68^Ga-PSMA PET/CT during their initial staging workup are found to have nodal metastases, with rates of N1 and M1a disease at 20% and 9%, respectively [3,4,5]. In patients with biochemically recurrent disease, either post-operatively or post-radiotherapy, ^68^Ga-PSMA PET/CT positivity rates of 32% for pelvic nodes, 14% for extra-pelvic nodes, 16% for bone metastases, and 1% for visceral metastases have been recorded, with positivity rates ranging from 48% at PSA 0.2–0.5 ng/mL to >90% at PSA > 2 ng/mL [6].

Elective nodal irradiation (ENRT) in the definitive treatment of prostate cancer has historically been a long-standing subject of controversy, and is further influenced by patient selection, radiation technique, and treatment volume [7,8]. Recent trials such as PIVOTAL and POP-RT support the safety and feasibility of ENRT [9,10]. A 2019 multi-institutional retrospective analysis and the phase 2 PEACE V-STORM and OLIGOPELVIS (GETUG-P07) trials further support ENRT as a possible standard treatment approach over metastasis-directed therapy (MDT) in the recurrent setting. They demonstrated improvements in nodal recurrence rates, metastasis-free survival and disease-free survival, while elucidating the para-aortic nodes as the major site of relapse after pelvis-only treatment [11,12,13,14].

Pencil-beam therapy (PBT) has the potential to confer several advantages over intensity-modulated radiotherapy (IMRT) or volumetric modulated arc therapy (VMAT) in treatment of pelvic/para-aortic nodes and in the re-irradiation setting, including low entrance dose, minimal exit dose beyond the Bragg peak, and lower integral dose, which results in reduced amounts of radiation being absorbed by the healthy tissues along the beam path. Several dosimetric studies comparing IMRT/VMAT to PBT for prostate-only treatment have demonstrated reductions in intermediate doses to bladder and rectal volumes using PBT [15,16,17]. Intensity-modulated proton therapy (IMPT) is preferred in treating pelvic and para-aortic lymph nodes, conferring reduced doses to organs-at-risk such as the duodenum, kidneys, liver, small and large bowels, bladder, and penile bulb [18,19,20,21]. This reduction in dose to the healthy tissues and organs at risk has the potential to lower risks of gastrointestinal, genitourinary, and sexual toxicities while decreasing risk of secondary malignancy [18]. The benefits of proton therapy for nodal re-irradiation include safer delivery of definitive doses, ability to dose-escalate when needed, and reduced cumulative normal tissue exposure [22,23]. While previous studies have focused on de novo radiotherapy treatment, none have detailed the dosimetric outcomes and toxicities of patients receiving re-irradiation using IMPT for prostate cancer with pelvic or para-aortic recurrence. Here, we evaluate the dosimetric profiles of men treated with IMPT, as compared with IMRT/VMAT plans, after their having previously received radiotherapy to the prostate and pelvic/para-aortic lymph nodes.

## 2. Materials and Methods

We conducted an institutional review board-approved retrospective analysis of 15 patients with isolated pelvic and/or para-aortic nodal recurrence of prostate cancer who received comprehensive nodal irradiation to the pelvic and/or para-aortic nodal chains, using VMAT or IMPT, at our institution between February 2020 and January 2026. Patients had histologically confirmed prostate adenocarcinoma and had undergone evaluation by complete history, physical examination, bone scintigraphy, and computer tomography (CT) of the abdomen and pelvis, PSMA PET-CT, and/or pelvic magnetic resonance imaging (MRI). All patients had previously received radiotherapy either to the intact prostate or the prostatic fossa, with or without irradiation of the pelvic lymph nodes. Patients were initially treated with brachytherapy (n = 2; 13.3%), stereotactic body radiotherapy (SBRT) (n = 1; 6.7%), PBT (n = 2; 13.3%), or IMRT/VMAT (n = 10; 66.7%). Table 1 details the patient characteristics, including their initial treatment fields and modalities, the time interval between their initial courses and their re-irradiation courses, and nodal disease burden, as shown on their PSMA PET-CTs. Patients were monitored weekly during treatment and generally seen 3–6 months after re-irradiation, with subsequent follow-ups at 6-month intervals. All patients received oral relugolix during re-irradiation.

### 2.1. Initial Treatment Planning and Delivery

Planning and delivery of both the original treatments and the re-irradiation courses were conducted using daily image-guided RT with kV and/or cone-beam computed tomography (CBCT) imaging. All organs-at-risk (OARs) volumes and dose constraints, as well as the clinical (CTVs) and planning target volumes (PTVs), were delineated in accordance with the RTOG 0534 protocol and NRG Oncology Consensus Pelvic Lymph Node Atlas [24,25].

IMPT plans were generated using the Raystation v.2023B treatment planning system (RaySearch Laboratories, Stockholm, Sweden) and used pencil beam scanning with discrete spot scanning. Multiple-field optimization (MFO) was used to deliver appropriate variations in dose from each field, prioritized to spare the bowel cavity, sigmoid colon, rectum, bladder, penile bulb, and femoral heads. For image registration, rigid registration was conducted between the initial and re-irradiation CT simulation images, followed by a deformable registration using the correlation coefficient and mutual information algorithms within Raystation. This hybrid deformable registration was then used as the frame for dose-mapping.

All IMPT plans combined expansion and robust optimization with 3.5% range uncertainty and 5 mm setup uncertainty from CTV, with alignment to bone. For the original plans, prescription doses to the whole pelvis and prostate bed PTVs from the initial VMAT plans were 45–50.4 Gy and 66.6–72 Gy, respectively, in 1.8–2 Gy (RBE) fractions. Treated IMPT re-irradiation prescription doses were 45–53.75 Gy radiobiological equivalent (GyE) to at-risk pelvic and para-aortic nodal chains and 60–64 GyE to gross nodal disease in 1.8–2 GyE fractions; proton doses were calculated using radiobiological effective doses of 1.1.

### 2.2. Treatment Re-Planning and Comparison

**Figure 1:**
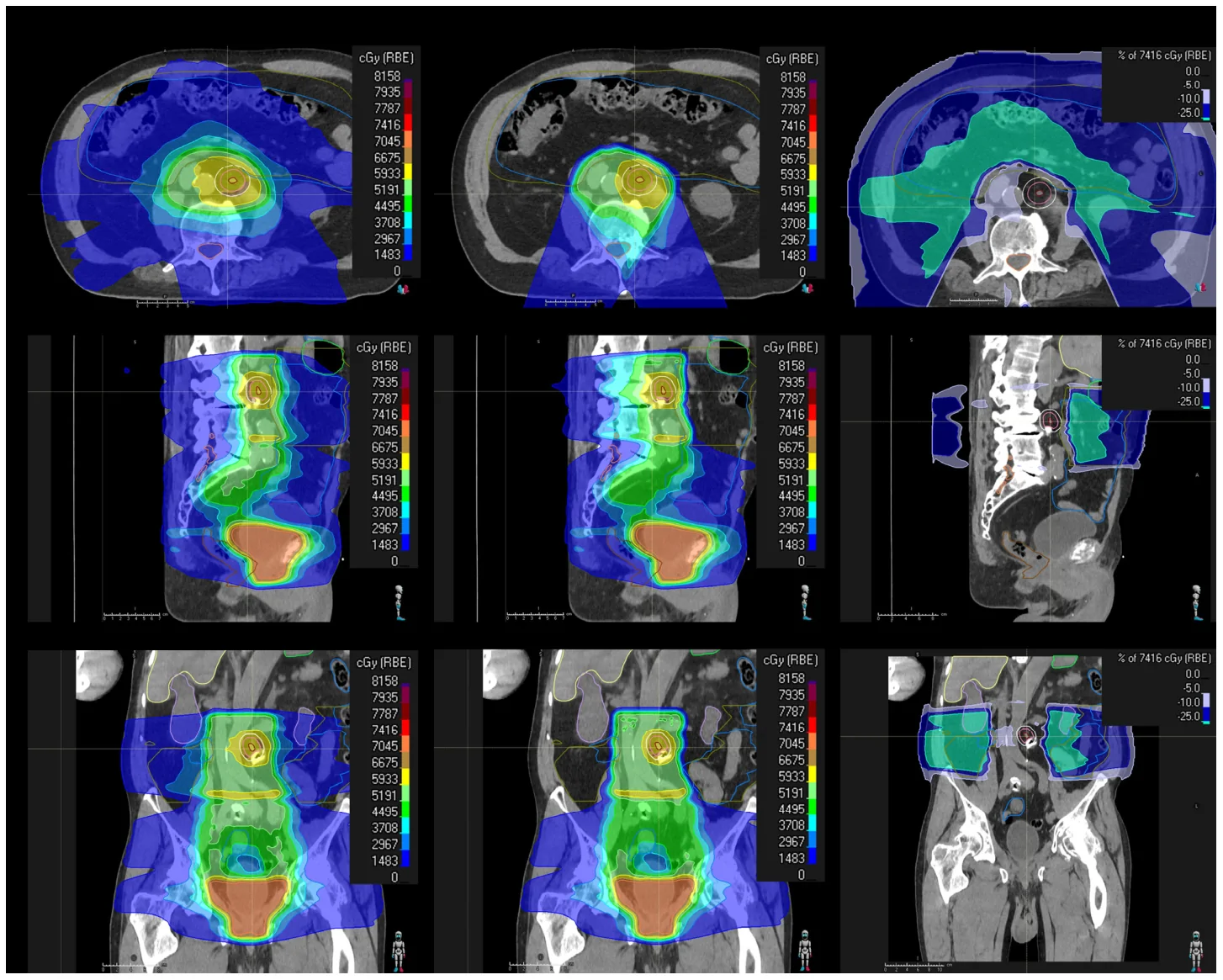
Axial, sagittal, and coronal views of composite-plan color washes, comprising the original plans, combined with either VMAT or IMPT re-irradiation plans targeting a para-aortic nodal recurrence. Differences in intermediate and low-dose baths are shown in the right column. OP = Original plan; VMAT = Volumetric modulated arc therapy; IMPT = Intensity-modulated proton therapy.

For re-planning and comparative study purposes, the CTV prescription doses were standardized for the regional pelvic and para-aortic nodes (CTV_50.4) and grossly involved nodal disease (CTV_63). Re-planned treatments were calculated to deliver 28 fractions at 1.8 Gy (RBE) fractions to the at-risk nodal chains and a simultaneous integrated boost (SIB) of 2.25 Gy (RBE) fractions to the gross nodal disease. The PTVs were used solely for dose comparison and reporting. OAR contours for all 15 patients were standardized by appropriate personnel (J.A.K.II, A.T., B.F.). VMAT and IMPT re-irradiation plans were both generated on Raystation v.2023B. VMAT plans consisted of two to three 360-degree coplanar arcs, and IMPT plans consisted of 3–4 beams, arranged either posteriorly or posterolaterally, depending on the inferior extent of the target. An example of the dose-color-wash distribution of each composite plan can be visualized in Figure 1.

Because standard fractionation at 1.8–2.0 Gy/fraction was used in both the original and re-irradiation plans, the doses from both plans were summed together to determine the cumulative dose received by each OAR, and a composite analysis of the original plans and the re-irradiation plans was generated. Of the 15 patients, four patients were excluded from the composite analysis: 2 (13.3%) patients, including one patient initially treated with CyberKnife, were treated at outside institutions and did not have retrievable records, and 2 (13.3%) patients had received brachytherapy as initial monotherapy, which would have precluded a more accurate comparison against those who had received EBRT. Therefore, 11 (73.3%) patients were included in the composite analysis.

### 2.3. Statistical Analysis

Target goals and OAR constraints used for comparison are shown below in Table 2. Maximum (Dxcc), mean (D_mean), and volumetric (VxGy) doses received by bladder, left and right femoral heads, left and right kidneys, left and right femoral heads, and infield (defined as OAR volume within 1 cm of CTV) rectum, sigmoid, bowel cavity, and body-CTV were appropriately recorded. Infield body was defined as the body volume 1 cm superior or inferior to the PTV displayed in the re-irradiation plans. Clinical goals were compared, using the paired, two-sided Student’s *t*-test, in Excel Version 16.74 (Microsoft, Redmond, WA, USA), with a *p* value of <0.05 considered significant. DVH-based comparisons of target coverage and OAR dose were conducted using Matlab R2025a (MathWorks, Natick, MA, USA). For target coverage, a right-tailed *t*-test was conducted to assess our hypothesis that IMPT provides higher coverage than VMAT. For OAR dose, a left-tailed *t*-test was conducted to assess our hypothesis that IMPT delivers a lower dose than VMAT. In our assessments of both target coverage and OAR dose, a *p* value of <0.05 was considered statistically significant.

## 3. Results

Table 2 provides comparative mean values for target coverage goals and OAR dose constraints for the nominal IMPT vs. VMAT plans, and the composite plans involving the original radiotherapy treatments and the IMPT vs. VMAT re-irradiation plans.

### 3.1. DVH Comparison for Targets

CTV_50.4 and CTV_63 coverages were met for all re-irradiation plans, with 99% of CTVs receiving ≥100% of prescription doses. There were no significant differences in CTV or PTV coverage between plans.

### 3.2. DVH Comparison for OARs in Nominal Re-Irradiation Plans

**Figure 2:**
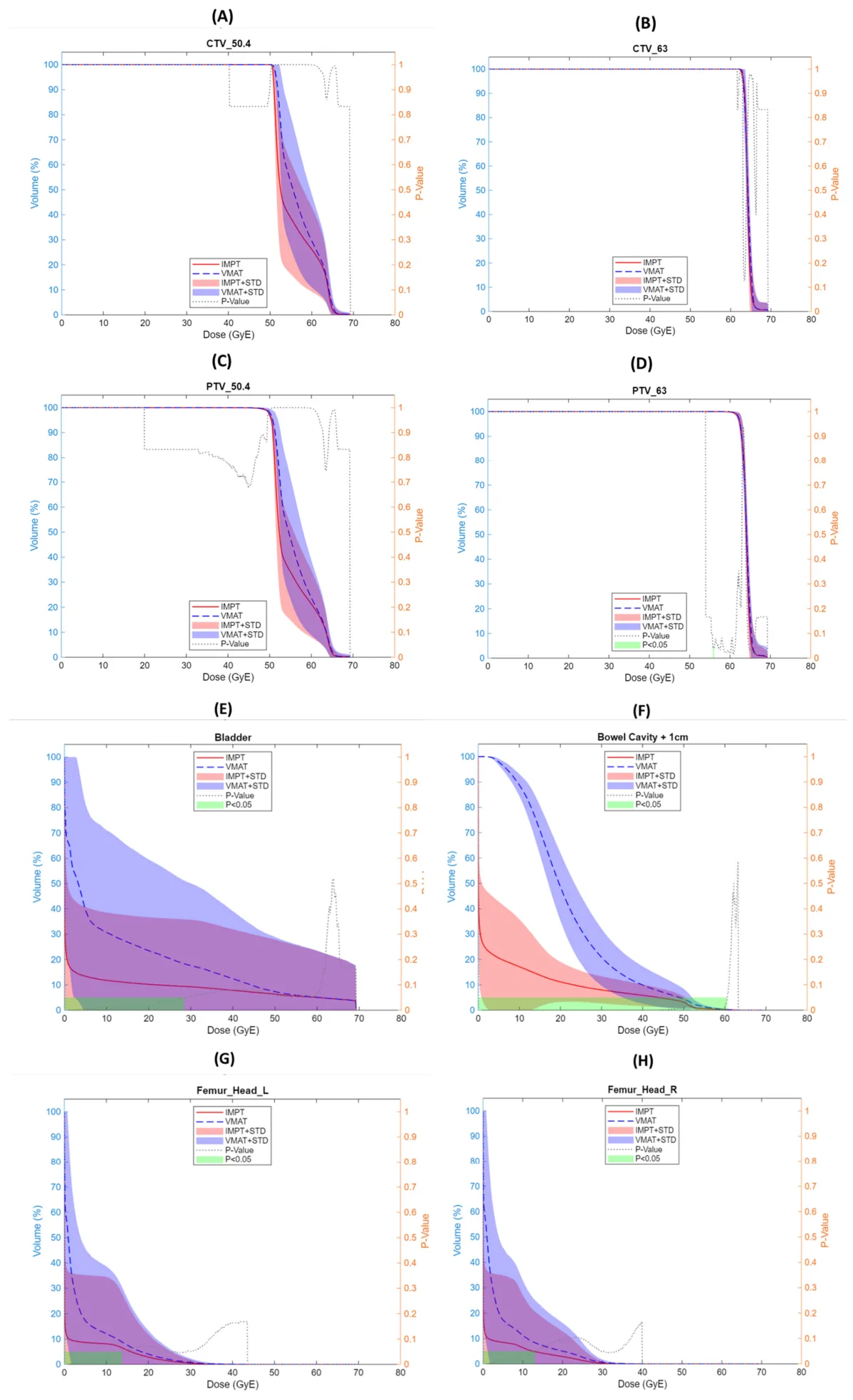

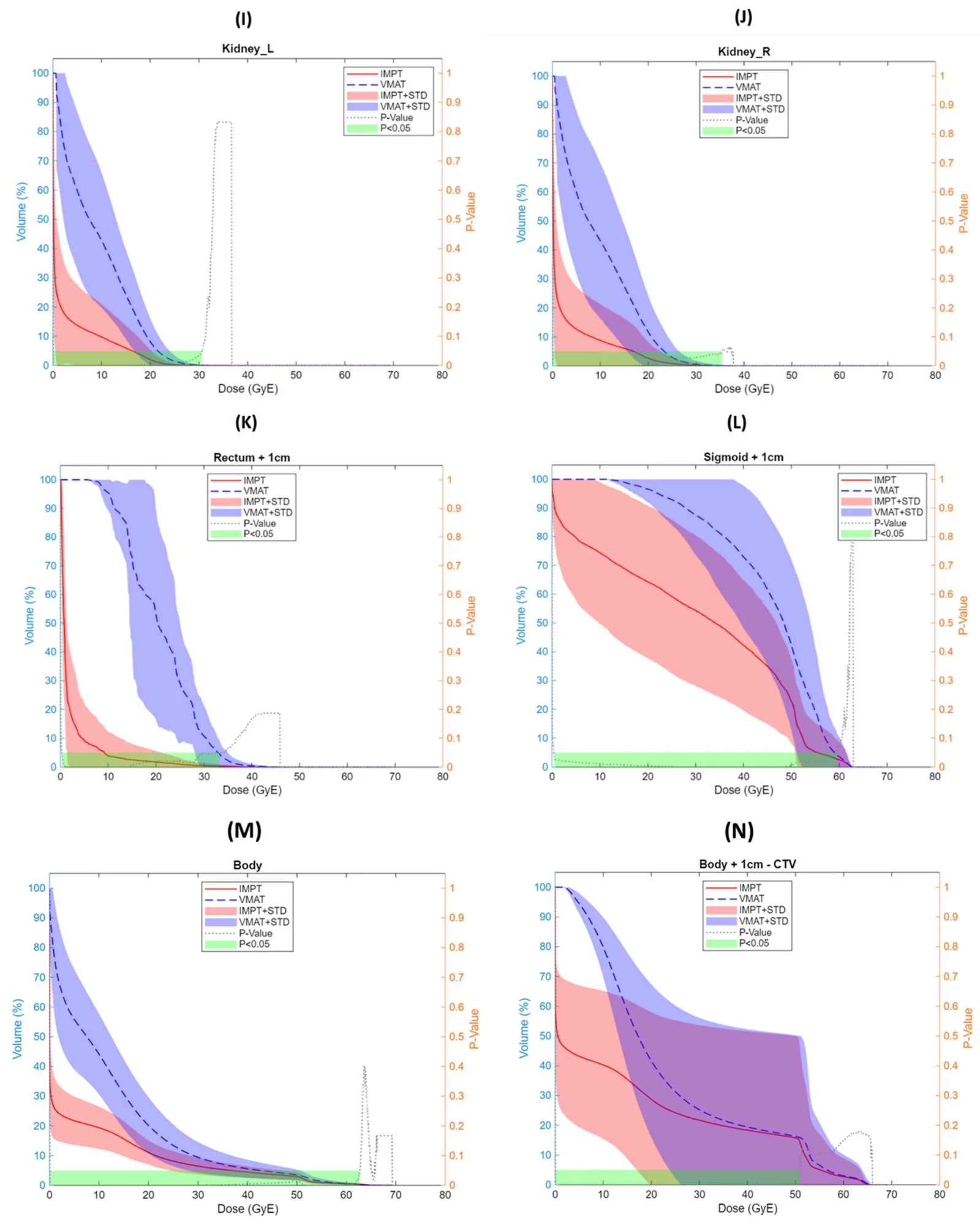
Comparison of VMAT and IMPT re-irradiation plans for each target and organs-at-risk (OARs). Doses to OARs shown over the entire DVH, comparing intensity-modulated proton therapy (IMPT) (red) and volumetric modulated arc therapy (VMAT) (blue), including CTV_50.4 (**A**), CTV_63 (**B**), PTV_50.4 (**C**), PTV_63 (**D**), Bladder (**E**), Bowel Cavity + 1 cm (**F**), Femur_Head_L (**G**), Femur_Head_R (**H**), Kidney_L (**I**), Kidney_R (**J**), Rectum + 1 cm (**K**), Sigmoid + 1 cm (**L**), Body (**M**), and Body + 1 cm—CTV (**N**).

Figure 2 shows the DVH metrics of the nominal IMPT vs. VMAT re-irradiation plans. The solid red line and dotted blue line indicate the mean values of the target coverages and OAR doses for IMPT and VMAT, respectively, and the shaded regions indicate standard deviations (STD). Dosimetric values that achieve statistical significance are included within the green shaded region. All OAR constraints were met with VMAT plans except for bowel cavity V15Gy and V55Gy; however, all dose constraints were met with the IMPT plans. IMPT plans generally achieved lower doses to OARs. The bowel cavity V15Gy (274.8 cc vs. 2112.8 cc, *p* < 0.001), V45Gy (116.8 cc vs. 172.4 cc, *p* < 0.001), V55Gy (16.1 cc vs. 37.4 cc, *p* < 0.001), and V60Gy (6.3 cc vs. 8.5 cc, *p* = 0.025) all saw significant reductions with IMPT. Bladder D0.03cc was lower for IMPT plans compared to VMAT (16.01 Gy vs. 21.04 Gy, *p* = 0.032). Left (6.73 Gy vs. 11.63 Gy, *p* = 0.006) and right (6.85 Gy vs. 11.66 Gy, *p* = 0.004) femoral head D0.03cc were lower with IMPT compared to VMAT. Left kidney V18Gy (2.69% vs. 13.83%, *p* = 0.001), left kidney D_mean (1.68 Gy vs. 9.07 Gy, *p* < 0.001), right kidney V18Gy (3.2% vs. 17.55%, *p* = 0.001), and right kidney D_mean (1.82 Gy vs. 9.54 Gy, *p* < 0.001) were all significantly reduced using IMPT. Rectum D0.5cc (2.95 Gy vs. 11.11 Gy, *p* = 0.008) was lower with IMPT, as well as sigmoid D0.03cc (30.31 Gy vs. 31.27 Gy, *p* = 0.011), V45Gy (5.2 cc vs. 10.5 cc, *p* = 0.008), and V55Gy (0.7 cc vs. 2.8 cc, *p* = 0.026). Finally, IMPT yielded a significant reduction in body-CTV integral dose (110.1 J vs. 237.7 J, *p* < 0.001), compared to VMAT.

### 3.3. DVH Comparison for Composite Plans

**Figure 3:**
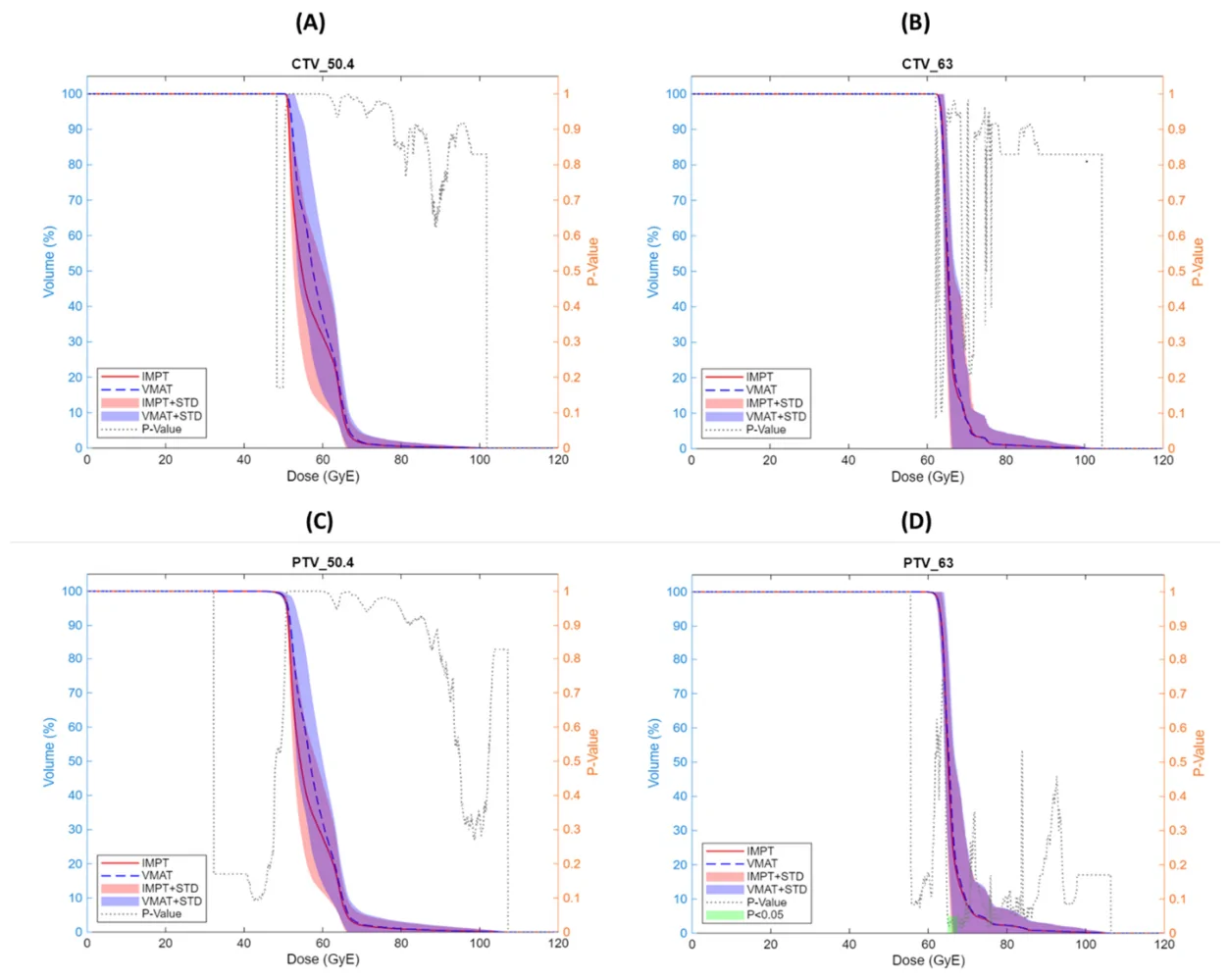

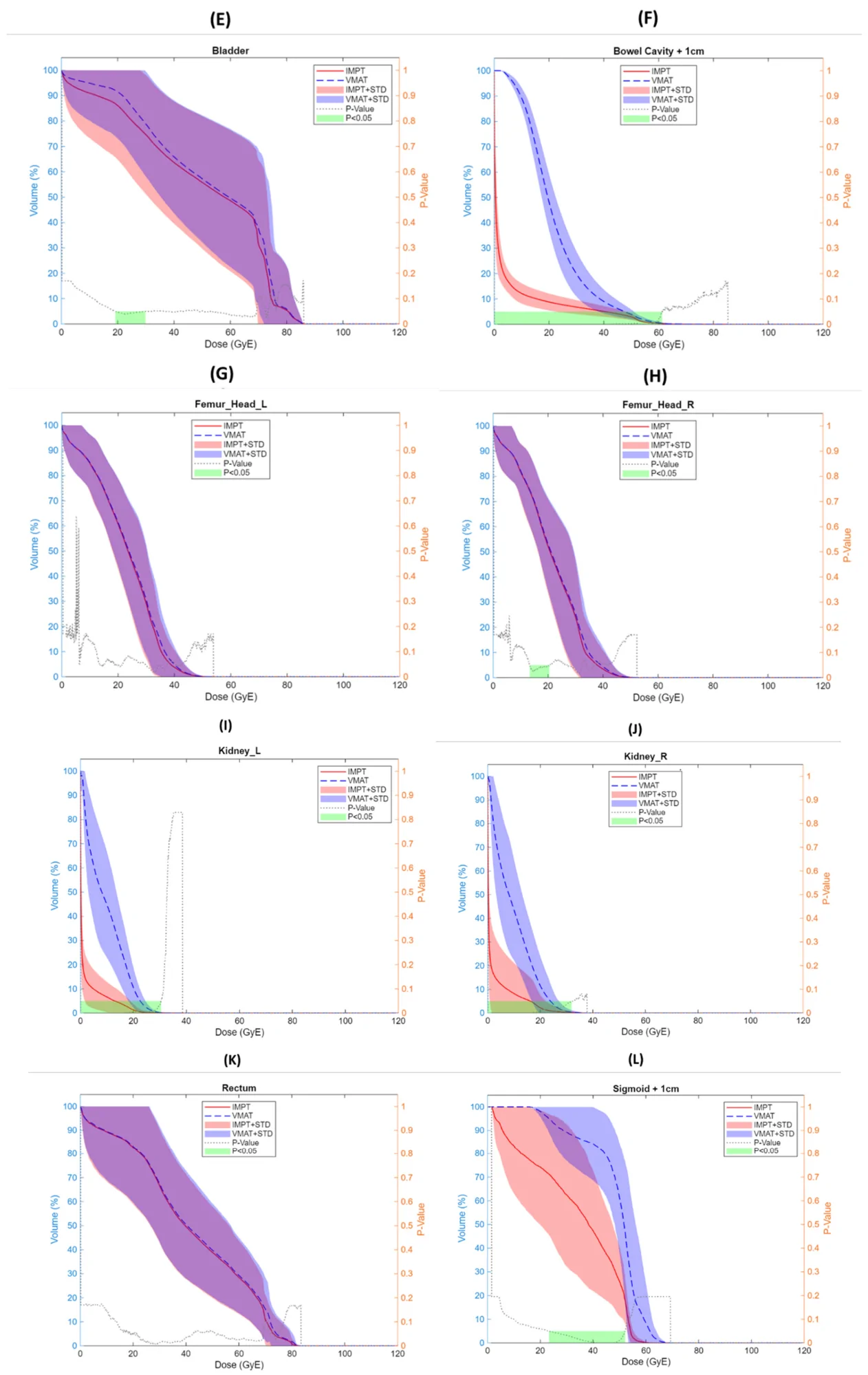

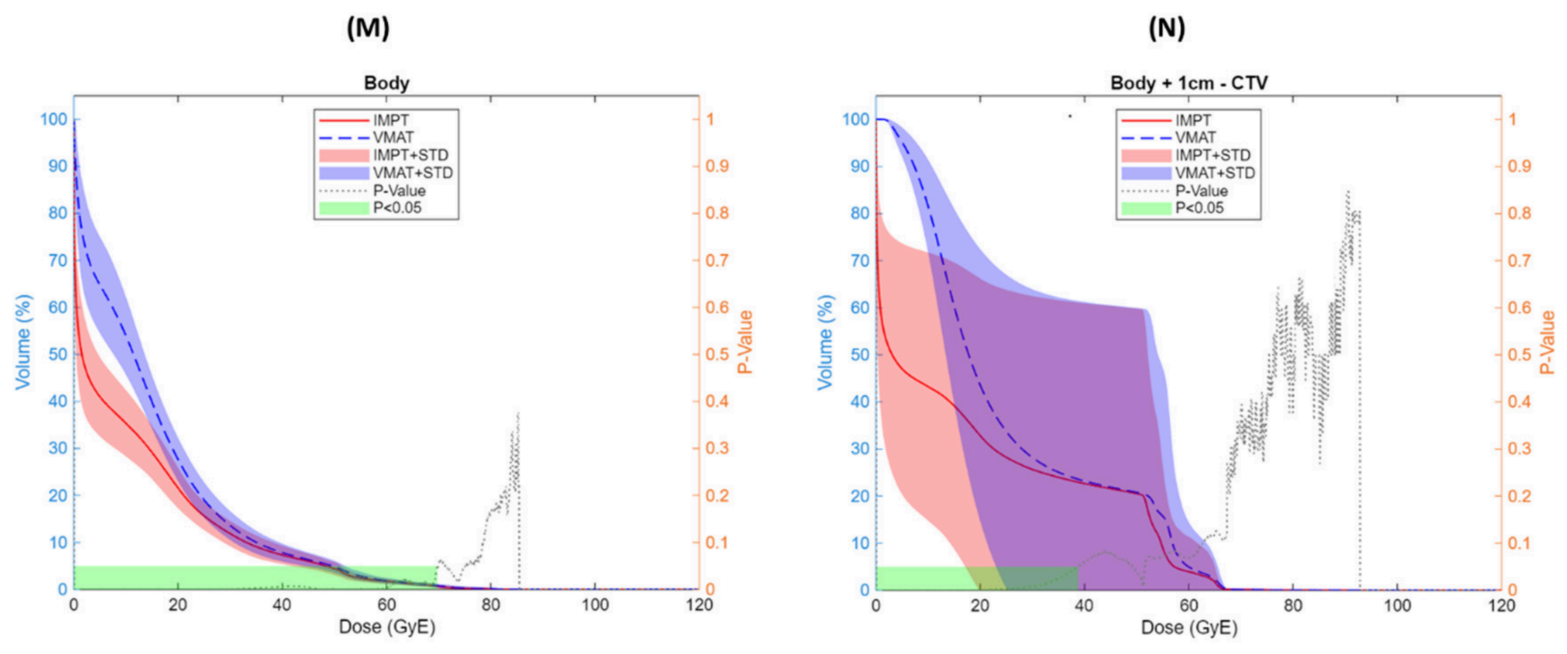
Composite analysis of the original EBRT plans combined with IMPT (OP + IMPT) vs. VMAT (OP + VMAT) re-irradiation plans, showing target coverage and dose to organs at risk. Doses to organs at risk shown over the entire DVH, comparing intensity-modulated proton therapy (IMPT) (red) and volumetric modulated arc therapy (VMAT) (blue), including CTV_50.4 (**A**), CTV_63 (**B**), PTV_50.4 (**C**), PTV_63 (**D**), Bladder (**E**), Bowel Cavity + 1 cm (**F**), Femur_Head_L (**G**), Femur_Head_R (**H**), Kidney_L (**I**), Kidney_R (**J**), Rectum (**K**), Sigmoid + 1 cm (**L**), Body (**M**), and Body + 1 cm—CTV (**N**).

Figure 3 shows the DVH values for the composite plans, including the prior radiation course. Annotations are the same as those in Figure 2. Dosimetric values that achieve statistical significance are included within the green shaded region. The composite IMPT plan conferred a statistically significant reduction in both bladder V65Gy (44.92% vs. 45.99%, *p* = 0.034) and D0.03cc (75.46 Gy vs. 77.17 Gy, *p* = 0.029). The bowel cavity V15Gy (593.7 cc vs. 2433.3 cc, *p* < 0.001), V45Gy (173.4 cc vs. 238.9 cc, *p* < 0.001), V55Gy (35.3 cc vs. 62.3 cc, *p* < 0.001), and V60Gy (17.3 cc vs. 24.1 cc, *p* = 0.021) were lower in the IMPT composite plans. The composite analysis of the left femoral head V37Gy (6.99% vs. 9.15%, *p* = 0.041) and Dmax (45.70 Gy vs. 46.77 Gy, *p* = 0.018) also showed dose reductions with IMPT. The right femoral head Dmax reduction (43.91 Gy vs. 44.94 Gy, *p* = 0.019) achieved significance with IMPT. Reductions in left kidney V18Gy (2.46% vs. 16.75%, *p* = 0.002), left kidney D_mean (1.70 Gy vs. 9.61 Gy, *p* < 0.001), right kidney V18Gy (3.75% vs. 16.66%, *p* = 0.003), and right kidney D_mean (2.13 Gy vs. 9.71 Gy, *p* < 0.001) all remained significant with IMPT on composite analysis. IMPT also conferred reductions in rectum D0.5 cc (74.10 Gy vs. 75.14 Gy, *p* = 0.019), V40 Gy (49.62% vs. 50.45%, *p* = 0.032), and V60Gy (28.48% vs. 29.27%, *p* = 0.026), respectively. There were no statistically significant differences in in-field sigmoid dosimetric values on composite analysis; however, the significant reduction in integral dose to Body-CTV was maintained (105.7 J vs. 211.7 J, *p* < 0.001) using IMPT compared to VMAT.

## 4. Discussion

This is the first study, to our knowledge, comparing pencil beam scanning IMPT against VMAT for re-irradiation of the pelvic and para-aortic lymph nodes for recurrent prostate cancer. The results demonstrated that IMPT confers significant reductions in maximum dose to bladder, femoral heads, rectum, sigmoid, and body integral dose, as well as mean and volumetric doses for bowel cavity, kidneys, and sigmoid. Of note, bowel cavity V15Gy and V55Gy metrics were exceeded with VMAT, but IMPT successfully respected these constraints under re-irradiation conditions. Composite analysis demonstrated that not only were the dosimetric benefits of IMPT re-irradiation sustained for all OARs except sigmoid, but proton therapy also conferred significant advantages.

Several volume-based metrics for bladder, femoral heads, rectum, and sigmoid did not experience a significant difference on composite analyses. These OARs were located mostly outside the re-irradiation field. In particular, the entire rectum was used in the composite analysis instead of the in-field rectum and rectum + 1 cm because all except one patient’s rectum + 1 cm volumes were outside the Body + 1 cm volume. However, our analysis showed the majority of dose contribution was from the original treatments and that re-irradiation with IMPT delivers less cumulative dose to the previously irradiated tissues.

### 4.1. Safety and Efficacy of Pelvic and Para-Aortic Nodal Re-Irradiation

Two prospective clinical trials, PLATIN-5 and PLATIN-4, assessed safety and feasibility of salvage pelvic lymph node radiotherapy (PLNRT) after radical prostatectomy alone (PLATIN-4) or prostate bed radiotherapy (PLATIN-5). In PLATIN-4, the pelvic lymphatic drainage received a 51 Gy total dose in 1.5 Gy single doses, with an SIB to the involved nodes of up to a 61.2 Gy total dose in 1.8 Gy single doses and an additional SIB to the prostate bed of up to a 68 Gy total dose in 2.0 Gy single doses. In PLATIN-5, the pelvic lymphatic drainage received a 46.8 Gy total dose in 1.8 Gy single doses, with an SIB of up to 57.2 Gy total dose in 2.2 Gy single doses to the metastatic nodes, with the pelvic nodal fields abutting the previous prostate bed radiotherapy fields. Combined analysis of these two trials showed median progression-free survival of 66 months in PLATIN-4 and 39 months in PLATIN-5, with late grade ≥ 3 GU and GI toxicities of 4% at 24 months of follow-up [26]. These trials, in addition to the OLIGOPELVIS and PEACE V-STORM trials, demonstrate the clinical safety and feasibility of pelvic re-irradiation using IMRT, as summarized in Table 3.

While investigations into nodal re-irradiation using IMRT are ongoing, there is a lack of dosimetric data in the literature evaluating the use of protons in pelvic and para-aortic nodal re-irradiation. A retrospective treatment-planning study comparing robustly optimized IMPT (ro-IMPT) against three-dimensional conformal radiation therapy (3D-CRT), IMRT, and single-field optimization proton beam (SFO-PBT) therapies was conducted in 13 patients to assess toxicity risks for post-operative whole pelvic radiotherapy for gynecologic malignancies. Median estimated normal tissue complication probability (NTCP) values for acute hematologic toxicities and acute and late GI toxicities were 0.20, 0.94 and 0.58 × 10^−1^ in 3D-CRT; 0.19, 0.65 and 0.24 × 10^−1^ in IMRT; 0.04, 0.74 and 0.19 × 10^−1^ in SFO-PBT; and 0.06, 0.66 and 0.15 × 10^−1^ in ro-IMPT, respectively. These results indicated that ro-IMPT could reduce acute hematologic and late GI toxicity risks, particularly by reducing the dose to the bone marrow and bowel bag while maintaining adequate dose coverage to the CTV [27].

### 4.2. Correlation Between Dosimetric Outcomes and Toxicity

Prior IMRT studies have correlated reductions in bowel dose—specifically, V45Gy < 120 cc to individual loops and V45Gy < 195 cc to the entire potential space—with decreases in acute grade ≥ 3 gastrointestinal toxicity, namely, nausea/vomiting, which translates to improved quality of life and treatment tolerance [28,29,30]. Our results showed that not only was IMPT able to deliver reduction in bowel V45Gy on both nominal and composite analyses, but that it was able to respect re-irradiation dose constraints in a manner that may be more challenging to achieve using VMAT. A recent phase 3 trial comparing patient-reported outcomes in patients who received either photon or photon therapy for intermediate or low-risk prostate cancer found no differences in bowel function or urinary, sexual, or hormonal domains [31]. However, in patients with a more advanced disease that requires larger fields to cover the pelvic or para-aortic nodes, reductions in bowel dose using IMPT would be anticipated to provide more of these benefits, while mitigating chronic toxicity risks of stricture or fistula.

Both the left and the right kidneys in our study had significant decreases in V18Gy and mean dose using IMPT compared to VMAT, on both nominal and composite analysis. Prior studies and QUANTEC data have indicated that mean dose to kidney and volume of kidney receiving >20 Gy have been associated with renal injury, and mean dose of 15–18 Gy has been shown to correspond with <5% risk of chronic renal injury at 5 years [28,32]. These findings are particularly relevant, considering the increases in life expectancy in patients with advanced prostate cancer: a population cohort study of Swedish patients with de novo metastatic castrate-sensitive prostate cancer showed an increase in 5-year overall survival with the introduction of doublet therapy, from 26% from 2008–2012 to 35% from 2017–2020 [33]. Longer survival confers a greater need for cumulative dose reduction to the kidneys, leading to longer-term protection of renal function. Our study indicates that IMPT provides this advantage over VMAT, with V18Gy and D_mean reductions on both the nominal and composite plans.

Strictures of the rectum and sigmoid as long-term sequelae have been well-documented long-term toxicities [34]. Compliance with rectal dose constraints of V50Gy < 50%, V60Gy < 35%, V65Gy < 25%, V70Gy < 20%, and V75Gy < 15% has been shown to limit late grade ≥ 2 and grade ≥ 3 toxicity to <15% and <10%, respectively, with conventional fractionation [28]. The phase 3 PARCER randomized trial compared late toxicity rates of image-guided IMRT against those of 3D-CRT in cervical cancer patients undergoing post-operative radiotherapy. The results showed that IMRT conferred a lower 3-year cumulative incidence of grade ≥ 2 late GI toxicity (21.1% vs. 42.4%) [35]. NTCP models for GI toxicity developed from this study found that bowel V30Gy and V40Gy were consistent predictors of acute and late grade ≥ 2 diarrhea and persistent GI toxicity, and the authors recommended pushing these metrics as low as feasible to minimize toxicity risk [36]. Our study found minimal dose contributions to both rectum and sigmoid from both the nominal IMPT and VMAT re-irradiation plans, and the composite plans showed that not only did IMPT contribute less dose than VMAT, but that the original radiotherapy treatments contributed the vast majority of dose to these OARs compared to the re-irradiation plans. Reduced rectosigmoid dose may translate to lower risk of stricture and long-term bowel changes for patients undergoing reirradiation.

A national cohort study was conducted, comparing secondary malignancy risk in patients treated with IMRT vs. 3DCRT vs. proton beam radiotherapy (PBRT). In a pooled analysis across all tumor types, PBRT was associated with significantly lower second cancer risk compared to IMRT. Particularly among patients with >5 years of follow-up, secondary cancer risk after PBRT was significantly lower in patients who had primary tumors of the breast, head and neck, and prostate [37]. A retrospective cohort in Japan also reported a significantly lower risk of secondary cancer after prostate cancer treatment with carbon ion therapy, compared to photon therapy [38]. Compared to VMAT, our study found that cumulative integral dose to the body outside of the CTV was significantly reduced with IMPT. This can correlate to reduction in secondary cancer risk in younger patients, which would pose clear benefits in reduced morbidity and improved long-term quality of life.

### 4.3. Limitations

Our study has some limitations. Firstly, all VMAT re-irradiation plans were generated retrospectively for comparison against the IMPT plans. However, all the plans were reviewed and approved by appropriate personnel. Secondly, 9/15 (60%) plans from the prior courses were generated more than two years prior to our analysis. However, our institutional protocols for planning and treatment have remained consistent over time. In terms of image registration, we acknowledge that the accurate estimation of large anatomical changes, particularly in regions bordering the initial and re-irradiation fields, is a limitation of hybrid deformable registration. However, our institutional protocols determined this method was feasible as an estimation of the prior dose region. The lack of graded toxicity, patient-reported outcome data, and normal tissue complication probability analyses to facilitate assessment of predicted and actual toxicity rates, and their correlation with our dosimetric analysis, is another limitation. Finally, we acknowledge that our target coverage and pointwise DVH comparisons were exploratory in nature, with multiplicity and small sample size having potential impacts for our statistical power. Continued patient accrual and evaluation will confer more robust statistical power on our future analyses.

## 5. Conclusions

Compared with VMAT, IMPT delivered a significant reduction in dose to OARs in the nodal re-irradiation setting, all while maintaining excellent target coverage. Cumulative analysis of the initial treatment plans and the re-irradiation plans showed that IMPT maintained meaningful reductions in dose, potentially decreasing the risk of long-term toxicity in these OARs and providing a rationale for the utilization of IMPT in the re-treatment setting. Further clinical investigation, including toxicity evaluations, patient reported outcome data, and normal tissue complication probability analysis would facilitate a more thorough understanding of the relationships between these dosimetric advantages and potentially more favorable long-term clinical outcomes and toxicity management.

## Figures and Tables

**Table 1:** Characteristics of the patients who received proton re-irradiation for recurrent pelvic and para-aortic nodal disease.

Patient ID	Age	Race	TNM Stage at Initial RT	Initial RT Field	Initial RT Modality	Interval Between RT Courses (Months)	Sites of Involved Nodes (#)	Total # Involved Nodes
1	64	White	cT3aN1M0	Prostate, SVs, & pelvic LN	Proton	26	Para-aortic (10)	10
2	61	White	cT1cN0M0	Prostate	LDR	85	Para-aortic (2) L Common Iliac (2) R Common Iliac (3) R External Iliac (1)	8
3	77	Asian	pT2N0M0	Prostate fossa	VMAT	43	Common Iliac (2) R External Iliac (1)	3
4	67	White	cT1cN0M0	Prostate	HDR	12	R External Iliac (1) L External Iliac (2) L Internal Iliac (1) R Obturator (2)	6
5	75	White	cT2aN0M0	Prostate	CyberKnife	201	Para-aortic (7) L Common Iliac (3	10
6	69	Black	cT3bN0M0	Prostate, SVs, & pelvic LN	IMRT	136	Para-aortic (10) L Common Iliac (2) L Common Iliac (2)	14
7	73	White	cT1cN0M0	Prostate fossa	IMRT	84	Para-aortic (2)	2
8	70	White	pT3bN0R1	Prostate fossa	IMRT	41	R Common Iliac (4)	4
9	76	White	cT2aN0M0	Prostate fossa	IMRT	44	Para-aortic (1) L External Iliac (1)	2
10	70	White	pT3aN0M0	Prostate fossa	IMRT	34	Para-aortic (2) R Common Iliac (3) L Common Iliac (3)	8
11	70	White	pT3bN1M0	Prostate fossa & pelvic LN	IMRT	38	Para-aortic (4) R Common Iliac (9)	13
12	67	Black	pT2N1M0	Prostate fossa & pelvic LN	IMRT	66	Para-aortic (3)	3
13	70	Black	NA	Prostate/SVs	Proton	12	Para-aortic (4) L Common Iliac (1) R Common Iliac (2) R Internal Iliac (1) R External Iliac (1)	9

# = number of lymph nodes.

**Table 2:** Dose–volume histogram (DVH) comparisons of target coverage and dose to organs-at-risk for IMPT vs. VMAT re-irradiation. Abbreviations: OP = Original plan; VMAT = Volumetric modulated arc therapy; IMPT = Intensity-modulated proton therapy.

		Current Plan			Composite with Prior Radiation		
Target/OAR	Clinical Goal	IMPT Mean ± SD (95% CI)	VMAT Mean ± SD (95% CI)	*p*-Value	OP + IMPT Mean ± SD (95% CI)	OP + VMAT Mean ± SD (95% CI)	*p*-Value
CTV_50.4	V 49.90 Gy > 99.0%	99.97% ± 0.08% (99.93–100%)	100% ± 0% (—)	0.108	100%% ± 0% (—)	100% ± 0% (—)	1.000
CTV_50.4	V 46.80 Gy > 99.9%	100% ± 0% (—)	100% ± 0% (—)	1.000	100% ± 0% (0%)	100% ± 0% (0%)	1.000
CTV_63	V 62.37 Gy > 99.0%	99.66% ± 0.75% (99.25–100%)	99.95% ± 0.11% (99.89–100%)	0.080	99.98% ± 0.06% (99.94–100%)	99.98% ± 0.04% (99.95–100%)	0.500
PTV_50.4	V 49.39 Gy > 98.0%	98.83% ± 0.4% (98.61–99.05%)	99.09% ± 0.81% (98.64–99.53%)	0.122	99.22% ± 0.56% (98.84–99.59%)	99.25% ± 0.76% (98.74–99.75%)	0.457
PTV_50.4	V 45.36 Gy > 99.9%	99.94% ± 0.21% (99.83–100%)	99.97% ± 0.13% (99.90–100%)	0.344	99.99% ± 0.03% (99.97–100%)	99.94% ± 0.16% (99.83–100%)	0.129
PTV_63	V 59.85 Gy > 98.0%	99.54% ± 1.3% (98.82–100%)	99.85% ± 0.41% (99.62–100%)	0.104	99.94% ± 0.12% (99.86–100%)	99.96% ± 0.07% (99.92–100%)	0.196
PTV_63	V 56.70 Gy > 99.9%	99.82% ± 0.67% (99.45–100%)	99.94% ± 0.23% (99.81–100%)	0.153	100% ± 0% (—)	100% ± 0% (—)	1.000
Bladder	V 70.00 Gy < 15.0%	0% ± 0% (—)	0% ± 0% (—)	1.000	31.56% ± 33.21% (9.86–53.87%)	37.64% ± 30.98% (16.82–58.45%)	0.100
Bladder	V 65.00 Gy < 20.0%	0.01% ± 0.03% (0–0.02%)	0% ± 0% (—)	0.167	44.92% ± 28.21% (25.97–63.87%)	45.99% ± 27.67% (27.4–64.58%)	0.034 *
Bladder	V 50.00 Gy < 15.0%	0.53% ± 1.29% (0–1.24%)	1.43% ± 3.5% (0–3.37%)	0.070	55.81% ± 26.12% (38.26–73.35%)	57.25% ± 25.01% (40.45–74.05%)	0.053
Bladder	V 45.00 Gy < 40.0%	0.88% ± 2.02% (0–2.00%)	3.21% ± 7.99% (0–7.64%)	0.080	59.63% ± 24.99% (42.84–76.41%)	61.28% ± 23.61% (45.42–77.15%)	0.054
Bladder	D 0.03 cc < 66.15 Gy	16.01 Gy ± 26.34 Gy (1.42 Gy–30.6 Gy)	21.04 Gy ± 25.76 Gy (6.77 Gy–35.3 Gy)	0.032 *	75.46 Gy ± 5.18 Gy (71.98 Gy–78.95 Gy)	77.17 Gy ± 5.48 Gy (73.49 Gy–80.85 Gy)	0.029 *
Bowel Cavity	V 60.00 Gy < 10.0 cc	6.3 cc ± 4.6 cc (3.7 cc–8.9 cc)	8.5 cc ± 5.2 cc (5.7 cc–11.4 cc)	0.025 *	17.3 cc ± 13.5 cc (8.3 cc–26.4 cc)	24.1 cc ± 19.9 cc (10.8 cc–37.5 cc)	0.021 *
Bowel Cavity	V 55.00 Gy < 20.0 cc	16.1 cc ± 9.6 cc (10.8 cc–21.4 cc)	37.4 cc ± 22.8 cc (24.8 cc–50 cc)	<0.001 *	35.3 cc ± 17.6 cc (23.5 cc–47.2 cc)	62.3 cc ± 30.9 cc (41.5 cc–83 cc)	<0.001 *
Bowel Cavity	V 45.00 Gy < 195.0 cc	116.8 cc ± 40.5 cc (94.4 cc–139.3 cc)	172.4 cc ± 69.7 cc (133.8 cc–211 cc)	<0.001 *	173.4 cc ± 60.5 cc (132.7 cc–214 cc)	238.9 cc ± 95 cc (175.1 cc–302.8 cc)	<0.001*
Bowel Cavity	V 15.00 Gy < 830.0 cc	274.8 cc ± 85.5 cc (227.4 cc–322.2 cc)	2112.8 cc ± 706.8 cc (1721.4 cc–2504.2 cc)	<0.001 *	593.7 cc ± 256.4 cc (421.5 cc–766 cc)	2433.3 cc ± 423.2 cc (2149 cc–2717.6 cc)	<0.001 *
Bowel Cavity	D 0.03 cc < 63.00 Gy	62.87 Gy ± 0.96 Gy (62.34 Gy–63.4 Gy)	63.08 Gy ± 0.36 Gy (62.88 Gy–63.29 Gy)	0.132	75.51 Gy ± 15.03 Gy (65.4 Gy–85.6 Gy)	76.36 Gy ± 14.28 Gy (66.77 Gy–85.95 Gy)	0.212
Femur_Head_L	V 50.00 Gy < 1.0%	0% ± 0% (—)	0% ± 0% (—)	1.000	0.09% ± 0.21% (0–0.23%)	0.12% ± 0.23% (0–0.27%)	0.170
Femur_Head_L	V 45.00 Gy < 5.0%	0% ± 0% (—)	0.01% ± 0.03% (0–0.02%)	0.167	1% ± 2.17% (0–2.46%)	1.22% ± 2.23% (0–2.72%)	0.082
Femur_Head_L	V 37.00 Gy < 50.0%	0% ± 0% (—)	0.09% ± 0.31% (0–0.26%)	0.148	6.99% ± 9.88% (0.35–13.63%)	9.15% ± 10.92% (1.81–16.48%)	0.041 *
Femur_Head_L	D 0.03 cc < 53.00 Gy	6.73 Gy ± 11.92 Gy (0.12 Gy–13.33 Gy)	11.63 Gy ± 16.48 Gy (2.50 Gy–20.75 Gy)	0.006 *	45.70 Gy ± 6.56 Gy (41.30 Gy–50.11 Gy)	46.77 Gy ± 6.77 Gy (42.22 Gy–51.32 Gy)	0.018 *
Femur_Head_R	V 50.00 Gy < 1.0%	0% ± 0% (—)	0% ± 0% (—)	1.000	0.06% ± 0.21% (0–0.21%)	0.11% ± 0.25% (0–0.27%)	0.170
Femur_Head_R	V 45.00 Gy < 5.0%	0% ± 0% (—)	0% ± 0% (—)	1.000	0.81% ± 2.21% (0–2.29%)	1.06% ± 2.31% (0–2.61%)	0.082
Femur_Head_R	V 37.00 Gy < 50.0%	0% ± 0% (—)	0.05% ± 0.16% (0–0.14%)	0.102	5.89% ± 10% (0–12.61%)	7.05% ± 10.53% (0–14.13%)	0.052
Femur_Head_R	D 0.03 cc < 53.00 Gy	6.85 Gy ± 11.71 Gy (0.36 Gy–13.33 Gy)	11.66 Gy ± 16.52 Gy (2.52 Gy–20.81 Gy)	0.004 *	43.91 Gy ± 6.69 Gy (39.42 Gy–48.41 Gy)	44.94 Gy ± 6.97 Gy (40.26 Gy–49.62 Gy)	0.019 *
Kidney_L	V 18.00 Gy < 30.0%	2.69% ± 4.15% (0.39–4.99%)	13.83% ± 11.15% (7.66–20.01%)	0.001 *	2.46% ± 3.55% (0.08–4.85%)	16.75% ± 11.54% (9–24.51%)	0.002 *
Kidney_L	D_mean	1.68 Gy ± 1.48 Gy (0.86 Gy–2.5 Gy)	9.07 Gy ± 3.70 Gy (7.03 Gy–11.12 Gy)	<0.001 *	1.70 Gy ± 1.33 Gy (0.81 Gy–2.59 Gy)	9.61 Gy ± 3.39 Gy (7.33 Gy–11.89 Gy)	<0.001 *
Kidney_R	V 18.00 Gy < 30.0%	3.2% ± 7.31% (0–7.25%)	17.55% ± 16.35% (8.5–26.61%)	0.001 *	3.75% ± 8.23% (0–9.27%)	16.66% ± 14.37% (7.01–26.32%)	0.003 *
Kidney_R	D_mean	1.82 Gy ± 2.53 Gy (0.42 Gy–3.22 Gy)	9.54 Gy ± 4.53 Gy (7.03 Gy —12.05 Gy)	<0.001 *	2.13 Gy ± 2.83 Gy (0.23 Gy–4.03 Gy)	9.71 Gy ± 3.59 Gy (7.30 Gy–12.12 Gy)	<0.001 *
Rectum	V 70.00 Gy < 10.0%	0% ± 0% (—)	0% ± 0% (—)	1.000	10.93% ± 10.32% (3.99–17.86%)	15.3% ± 13.05% (6.53–24.07%)	0.105
Rectum	V 60.00 Gy < 20.0%	0% ± 0% (—)	0% ± 0% (—)	1.000	28.48% ± 15.2% (18.27–38.7%)	29.27% ± 15.66% (18.75–39.79%)	0.026 *
Rectum	V 50.00 Gy < 30.0%	0% ± 0% (—)	0% ± 0% (—)	1.000	38.89% ± 18.97% (26.15–51.64%)	39.78% ± 19.41% (26.74–52.82%)	0.060
Rectum	V 40.00 Gy < 40.0%	0% ± 0% (—)	0.05% ± 0.18% (0–0.15%)	0.167	49.62% ± 21.85% (34.94–64.3%)	50.45% ± 22.41% (35.4–65.51%)	0.032 *
Rectum	D 0.5 cc < 64.89 Gy	2.95 Gy ± 9.12 Gy (0 Gy —8 Gy)	11.11 Gy ± 14.99 Gy (2.81 Gy–19.42 Gy)	0.008 *	74.10 Gy ± 4.54 Gy (71.05 Gy–77.15 Gy)	75.14 Gy ± 4.70 Gy (71.98 Gy–78.29 Gy)	0.019 *
Sigmoid	V 60.00 Gy < 10.0 cc	0.3 cc ± 0.9 cc (0 cc–0.7 cc)	0.5 cc ± 1.2 cc (0 cc–1.1 cc)	0.103	2.8 cc ± 3.8 cc (0.3 cc–5.3 cc)	4.8 cc ± 6 cc (0.7 cc–8.8 cc)	0.081
Sigmoid	V 55.00 Gy < 20.0 cc	0.7 cc ± 1.6 cc (0 cc–1.5 cc)	2.8 cc ± 4.8 cc (0.1 cc–5.4 cc)	0.026 *	4.9 cc ± 6.2 cc (0.8 cc–9 cc)	8.5 cc ± 10.8 cc (1.3 cc–15.8 cc)	0.075
Sigmoid	V 45.00 Gy < 175.0 cc	5.2 cc ± 5.9 cc (1.9 cc–8.5 cc)	10.5 cc ± 12 cc (3.9 cc–17.2 cc)	0.008 *	18.8 cc ± 17.1 cc (7.3 cc–30.3 cc)	26.5 cc ± 25.8 cc (9.2 cc–43.9 cc)	0.090
Sigmoid	D 0.03 cc < 63.00 Gy	30.31 Gy ± 29.46 Gy (14 Gy–46.62 Gy)	31.27 Gy ± 29.01 Gy (15.20 Gy–47.33 Gy)	0.011 *	64.91 Gy ± 9.82 Gy (58.31 Gy–71.50 Gy)	66.31 Gy ± 11.07 Gy (58.87 Gy–73.74 Gy)	0.055
Body	D 0.03 cc < 67.41 Gy	65.74 Gy ± 0.48 Gy (65.47 Gy–66 Gy)	66.01 Gy ± 0.43 Gy (65.77 Gy–66.24 Gy)	0.016 *	83.08 Gy ± 13.61 Gy (73.94 Gy–92.23 Gy)	85.50 Gy ± 12.33 Gy 77.21 Gy–93.78 Gy)	0.033 *
Body-CTV	Integral Dose (J)	110.1 J ± 51.7 J (81.5 J–138.8 J)	237.7 J ± 123.1 J (169.5 J–305.9 J)	<0.001 *	105.7 J ± 49.2 J (72.6 J–138.7 J)	211.7 J ± 107.5 J (139.5 J–283.9 J)	<0.001 *

* = Considered statistically significant by *p* < 0.05.

**Table 3:** Prospective trials assessing safety and efficacy of pelvic nodal re-irradiation.

Study	Patients	mFU (mo)	Outcomes
OLIGOPELVIS (GETUG-P07) 2021 [13]	Group A (prior RP; n =27) Group B (prior RP with PB local relapse; n = 4) Group C (prior RP + PB-RT; n = 30) Group D (prior prostate RT; n = 6 (3 EBRT; 3 BT))	49.4	2Y-PFS: 81% 3Y-PFS: 58% mPFS: 45.3 mos 2Y-BRFS: 58% 3Y-BRFS: 46% mBRFS: 25.9 mos
PLATIN-4 2024 [26]	LN mets after RP who underwent PBRT and PLNRT with SIB (n = 39)	54	mPFS: 66 mos mOS: not reached
PLATIN-5 2024 [26]	LN mets after RP and PBRT who underwent PLNRT as field abutment with SIB to involved nodes (n = 39)	74	mPFS: 39 mos mOS: 117 mos
PEACE V-STORM 2025 [12]	Metastasis-directed therapy (MDT; n = 97) Elective nodal radiotherapy (ENRT; n = 93)	50	4Y-MFS: 63% vs. 76% (*p* = 0.063)

Key: BRFS = biochemical relapse-free survival; BT = brachytherapy; EBRT = external beam radiotherapy; ENRT = elective nodal radiotherapy; LN = lymph node; MDT = metastasis-directed therapy; OS = overall survival; RP = radical prostatectomy; PFS = progression-free survival; PB = prostate bed; PBRT = prostate bed radiation therapy; PLNRT = pelvic lymph node radiation therapy; SIB = simultaneous integrated boost.

## Data Availability

Research data are stored in an institutional repository and will be shared upon request to the corresponding authors.

## Abbreviations

The following abbreviations are used in this manuscript:

CT	Computed Tomography
CTV	Clinical Target Volume
DVH	Dose–Volume Histogram
EBRT	External Beam Radiotherapy
ENRT	Elective Nodal Irradiation
GyE	Gy Radiobiologic Equivalent
IMPT	Intensity-Modulated Proton Therapy
IMRT	Intensity-Modulated Radiotherapy
MDT	Metastasis-Directed Therapy
MFO	Multi-Field Optimization
MRI	Magnetic Resonance Imaging
OAR	Organs-at-Risk
OP	Original Plan
PBT	Pencil-Beam Therapy
PET	Positron Emission Tomography
PSMA	Prostate-Specific Membrane Antigen
PTV	Planning Target Volume
RBE	Relative Biological Effectiveness
SBRT	Stereotactic Body Radiotherapy
VMAT	Volumetric Modulated Arc Therapy
